# Lifestyles, Depression, Anxiety, and Stress as Risk Factors in Nursing Apprentices: A Logistic Regression Analysis of 1193 Students in Lima, Peru

**DOI:** 10.1155/2019/7395784

**Published:** 2019-11-06

**Authors:** Jessica Diaz-Godiño, Luz Fernández-Henriquez, Florencia Peña-Pastor, Patricia Alfaro-Flores, Gloria Manrique-Borjas, Frank Mayta-Tovalino

**Affiliations:** ^1^Nursing Professional School, Universidad Privada San Juan Bautista, Lima, Peru; ^2^Vice-Rectorate, Universidad Privada San Juan Bautista, Lima, Peru; ^3^Postgraduate Department in Stomatology, Universidad Científica Del Sur, Lima, Peru

## Abstract

Currently, it is considered that mental disorders are related to different types of chronic pathologies; for this reason, efforts to improve general health should also focus on preserving mental health. Therefore, the objective of this study was to determine through logistic regression if the independent variables (risk factors) such as (*X*1) age, (*X*2) sex, (*X*3) marital status, (*X*4) number of children, and (*X*5) occupation have influence on the dependent variables such as lifestyles, depression, anxiety, and stress in Peruvian nursing students. The research study was descriptive, transversal, and prospective; 1193 nursing students from Chorrillos, Ica, and Chincha were evaluated, which constituted the total population for the 2018 semester. The Health Promoting Life Profile-II (HPLP-II) and the Depression and Anxiety Stress Scale-21 (DASS-21) were used as instruments. 53.9% of nursing students had unhealthy lifestyles; however, they presented moderate (19.7%), slight (14.2%), severe (2.5%), and extremely severe (2.4%) anxiety. With respect to depression, it was found that 61.2% and 59.9% of affected students were stressed. A significant association was found only between depression and age (*p*=0.040) and OR = 2.0 (95% CI 1.3–3.1), anxiety and marital status (*p*=0.043) and OR = 1.7 (95% CI 1.0–2.6), and lifestyles and sex of the students (*p*=0.003) and OR = 1.1 (95% CI 1.1–2.3). Finally, it is concluded that Peruvian nursing students showed levels of anxiety ranging from moderate to extremely severe, while most of them had “normal” states of depression and stress and also showed unhealthy lifestyles.

## 1. Introduction

During the university stage, adolescents and young people have to take responsibility for their decisions, and these situations can become risk factors; they must also face the social and academic demands of their training [1]. For example, it is worth noting that mental and brain disorders are the biggest burden of disease in Europe, which not only affects the people who suffer from it, but also their caregivers and society in general because they cause an increase in costs for direct and indirect care and the attention they demand, which alters social spending and produces losses for personal development [2].

Disorders of mental origin are related to a variety of chronic pathologies; therefore, the effort to improve comprehensive health should also focus on improving mental health. Although, some research suggests that the training institution did not perform the function that could mean encouraging healthy behaviors and neglecting harmful behaviors [3, 4].

For the nursing professional of the primary health level, it is important to identify these study variables because it will favor establishing better educational strategies and preventive-promotional activities. This work was carried out in a coordinated manner and was based on the identification of factors linked to physical and mental health that could lead to the reduction of noncommunicable diseases, with the university students themselves assuming different lifestyles and adequate ways of coping with the mental health demands [5]. Achieving the well-being of university students requires care, especially considering that they have greater access to information; their lifestyles must contribute to the construction of ways of taking care of their integral balance, which stimulate health responsibility and culture, generating health-promoting behaviors among students, families, and the university community [6].

There is scarce literature describing the impact of mental disorders as risk factors for the academic performance of nursing students. Therefore, the purpose of the research was to determine lifestyles, depression, anxiety, and stress among the Peruvian nursing students.

## 2. Methods

### 2.1. Participants of the Study

A cross-sectional, observational study was conducted during 2018. It had the following dependent variables: lifestyles, depression, anxiety, and stress, while the independent variables (risk factors) were (*X*1) age, (*X*2) sex, (*X*3) civil status, (*X*4) number of children, and (*X*5) occupation. To demonstrate the hypothesis of the study that consisted in determining the influences of lifestyles on depression and anxiety in nursing students, the student population (*N* = 1193) enrolled in the I to X semester who strictly met the selection criteria was evaluated. The unit of analysis was formed by people studying in the Professional School of Nursing of Universidad Privada San Juan Bautista. Finally, this research was carried out following the guidelines of STROBE (strengthening the reporting of observational studies in epidemiology).

### 2.2. Selection Criteria

- Nursing students of both sexes, who agree to participate voluntarily in the study.
- Nursing students who attend regularly during the semester of study.
- Nursing students who were receiving pharmacological treatment for depression, anxiety, or stress at the time of the research were excluded.

### 2.3. Authorization of the Institution

A letter requesting authorization was presented to the Faculty of Health Sciences for the execution of the work in the Professional School of Nursing. We coordinated with the management of the Professional School of Nursing to determine the schedules for the application of the questionnaires in the different study cycles.

### 2.4. Calibration of Interviewers

To carry out the calibration of the interviewers, a pilot study was carried out (*n* = 100 students), where they were trained to act as a team during the application of the instrument; in this way, they were able to inform the participants using the same message, which ensured the complete filling of the questionnaires and the quality of the data. The calibration of the two pollsters was done using Cohen's kappa coefficient, and a value of 0.747 was obtained with *p* < 0.001.

### 2.5. Application of the Instrument

Prior to the delivery of the self-administered instruments, they were explained about their participation and confidentiality, to obtain their authorization by signing the informed consent. After obtaining the consent, the indications for filling out the following questionnaires were given: the HPLP-II (Health Promoting Life Profile-II) and the Depression and Anxiety Stress Scale-21 (DASS-21).

The Health Promoting Life Profile-II instrument (HPLP-II), authored by Nola Pender together with Walker and Sechrist in 1987, was applied. The same authors validated the Spanish version in 1990, with a reliability of 0.93. The HPLP-II instrument consists of 52 items subdivided into the following dimensions: nutrition, physical activity, responsibility in health, spiritual growth, interpersonal relationships, and stress management. These questions have a Likert scale for the answers, ranging from 1 to 4: never (1), sometimes (2), frequently (3), and routinely (4) [7].

The DASS-21 is an individual scale, composed of 21 questions that have answers ranging from 0 (nothing applies to me) to 3 (it applies a lot to me and most of the time). In addition, the instrument is subdivided into questions that determine depression, anxiety, and stress. The psychometric properties reported by Henry and Crawford [8] were 49% of explained variance, and a reliability was demonstrated through the Cronbach's alpha of 0.93 for all the items; this result was found with the application of the instrument in 1700 adults of a British population. In 2012 in Chile, they validated the Spanish version made by Antúnez y Vinet that reached a Cronbach's alpha of 0.91. They also verified that there were significant correlations between the DASS-21 and similar scales such as the Beck Depression Inventory-II and the Beck Anxiety Inventory [9].

### 2.6. Statistic Analysis

The results of the variables (lifestyles, depression, anxiety, and stress) were tabulated with the use of the Excel 2013 spreadsheet including personal data on age, gender, academic cycle, marital status, and occupation. The normal distribution was determined by the Kolmogorov-Smirnov test; subsequently, the Chi-square test was used to determine the respective associations; finally, the odds ratio (OR) was used to identify the risk factors. The level of significance established was *p* <0.05. All statistical analysis was performed using SPSS 23.0 software.

### 2.7. Ethical Aspects

To guarantee compliance with the bioethical principles, it was presented to the Ethics Committee of the Vice-Rector for Research of the San Juan Bautista Private University, obtaining approval with code no. 003-CEI-VRI-UPSJB. The research respected the process at all times, and the ethical principles related to working with people were followed as listed in the Belmont Report. The autonomy of nursing students was respected through informed consent, and the investigation did not imply any risk that endangers the health of the participants. In addition, each of them was treated with respect, not showing any type of discriminatory action, and giving each of the students the same opportunity to participate in the investigation.

## 3. Results

### 3.1. Study Population

According to the categories established in the respective instrument, it was found that in the evaluation of lifestyles, the majority of nursing students have unhealthy lifestyles in 53.9% (*n* = 643); likewise, the majority did not present depression at 61.2% (*n* = 730); however, moderate levels of 19.7% were found (*n* = 235), slight 14.2% (*n* = 169), severe 2.5% (*n* = 30), and extremely severe 2.4% (*n* = 29). Regarding anxiety, it was found that the highest prevalences were at the moderate level 36.3% (*n* = 433), in relation to stress, the majority were at normal levels 59.9% (*n* = 715) (Table 1).

### 3.2. Lifestyles

When evaluating the lifestyles of the nursing students, it was found that they were not healthy, mainly in students who were between 20 and 29 years of age with 29.7% (*n* = 354); female students were the most frequent 86.2% (*n* = 1028). Regarding the marital status of the students, it was found that the most frequent was the category of singles 87.1% (*n* = 1039). When performing the logit model to determine if the variables (*X*1) age, (*X*2) sex, (*X*3) marital status, (*X*4) number of children, and (*X*5) occupation are directly influencing the lifestyles of undergraduate students of nursing, it was evidenced that only the variable (*X*2) sex was a risk factor with an adjusted OR = 1.6 (95% CI 1.1–2.3) and *p*=0.003 (Table 2).

### 3.3. Depression

When evaluating depression in nursing students, the normal category was found in the majority of the students from 20 to 29 years of age, 34.6% (*n* = 413), followed by moderate depression in 10.6% (*n* = 127), mild in 7.0% (*n* = 83), severe in 1.5% (*n* = 18), and extremely severe in 1.1% (*n* = 13). When evaluating the risk factors (independent variables) in the logit model to determine if the variables (*X*1) age, (*X*2) sex, (*X*3) marital status, (*X*4) number of children, and (*X*5) occupation are directly influencing the depression of undergraduate nursing students, it was evidenced that only the variable (*X*1) age was a risk factor with an adjusted OR = 2.0 (95% CI 1.3–3.1) and *p*=0.040 (Table 3).

### 3.4. Anxiety

It was found that the majority had a moderate level, with 19.4% (*n* = 232), normality 13.6% (*n* = 162), and mild 11.6% (*n* = 138) among students from 20 to 29 years old. Female students showed moderate (31.1% (*n* = 371)), normal (20.1% (*n* = .240)), and mild (19.9% (*n* = 238)) anxiety. However, assessing risk factors (independent variables) in the logit model to determine whether the variables age (*X*1), sex (*X*2), marital status (*X*3), number of children (*X*4), and occupation (*X*5) can directly influence the anxiety of nursing undergraduate students. It was shown that all these variables were not a risk factor (Table 4).

### 3.5. Stress

According to the categories established in the instrument, stress (state of mental fatigue caused by the demand for a performance much higher than normal) in nursing students was found in most of them at normal levels (32.4% (*n* = 386)), especially in those between 20 and 29 years of age, moderate level (8.0% (*n* = 95)), and slight level (8.4% (*n* = 100)). When establishing the logistic regression model of the risk factors of the students of the Professional School of Nursing according to the level of stress, it was found that the variables age, sex, civil status, number of children, and occupation were not statistically significant in the influence on the stress of nursing students. In addition, all these variables did not represent a risk factor (Table 5).

## 4. Discussion

In different parts of the world, people are affected by their health as a consequence of inadequate behaviors regarding their health care, that is to say, daily lifestyles that in the medium or long term will cause deterioration of their health [10]. On the contrary, mental health problems require early detection, especially at an early age, for better management and treatment, which is why this research aims to provide specific information about this population through its results.

The early detection of mental problems and the determination of lifestyles in nursing students are important for what can originate as a consequence at the academic level and the need to establish timely interventions and reduce the impact of these situations on the environment of the university [11]. This study aimed to describe and specify important characteristics related to the health of nursing students; for this purpose, the data collection was carried out using the survey technique and instruments such as Health Promoting Life Profile-II (HPLP-II) the authorship of Pender, Walker, and Sechrist (1987), which was used in the study by Hosseini et al. [12] in 2013 in a sample of 404 nursing students from the University of Tehran, and in the same way by Polat et al. [13] in nursing students during 2012 to 2013. This instrument has a high reliability both in its original version and in its Spanish version; it has been shown that this instrument has high reliability, and it has also been used in several countries, especially in students of Health Sciences (Nursing). 

For the measurement of depression, anxiety, and stress, we used the Depression and Anxiety Stress Scale-21 (DASS-21) questionnaire, which was applied in the studies of Cheung et al. [14] in 661 nursing students from the University of Hong Kong; likewise was applied in the study of Shamsuddin et al. [15] in 506 university students from Malaysia. This type of scale has been evaluated by different authors who have demonstrated their adequate psychometric ability and with high reliability. The study of validity made in Chile for its Spanish version was made in adolescent students and university students, and verified its effective application in this population group.

In this study, it was determined that Peruvian nursing students had unhealthy lifestyles 53.9%, did not show depression 61.2%, and stress 59.9%, but moderate anxiety levels 36.3%. These data can be contrasted with those found by Shamsuddin et al. [15] who evidenced that the prevalence of anxiety was higher compared to depression and stress; in the same way, this coincides with the results of Cheung et al. [14] where they also showed that anxiety was more prevalent with 39.9%. 

Unhealthy lifestyles were found in the majority of nursing students from 20 to 29 years of age, female, single, who did not have children, and those who only studied. There was also a significant difference in lifestyles with the sex (*p*=0.003) and an OR = 1.661. In relation to these results, Hosseini et al. [12] found that students who were married had a better health promotion behavior; however, Almutairi et al. [16] found that gender was a predictive factor for unhealthy styles in students from Saudi Arabia. There are also coincidences with the study by Rizo-Baeza et al. [17] who found moderate lifestyles in students. On the contrary, Daesy et al. [18] found that students had risk behavior that included alcohol consumption, incorrect diets, and poor physical activity. Also, they differ from that found by Mak et al. [19] who found that students did not engage in risky behaviors regarding their health.

Regarding depression, the majority of students presented a normal state although levels of mild, moderate, severe, and extremely severe depression were found in a lower proportion in those students between 20 and 29 years of age, female, single marital status without children, and who only dedicate themselves to study. A correlation was found between the students' age and depression, and it was also statistically verified that it constitutes a risk factor for suffering depression. The results related to depression are similar to the study by Shamsuddin et al. [15] because they found among their findings that depression and anxiety were higher in students 20 years and older; likewise, Abebe et al. [20] found that depression (19.1%) and anxiety (23.6%) were associated with the sex of the students. Likewise, Hamaideh et al. [21] found in their study that the levels of depression were mild and that in addition those who suffered were women, unlike Cheung et al. [14] who found that depression levels were moderate to extremely severe in 24.3% of nursing students.

On the other hand, anxiety occurs in nursing students at moderate to extremely severe levels, which occurs in those who are between 20 and 29 years old, women, single women, who do not have children, and who are dedicated only to study. Among the results obtained, it was found that marital status is significantly associated with the presence of anxiety and that sex constitutes a risk factor for this type of disorder. This is similar to that found by Hamaideh and Hamdan-Mansour [21] who found that anxiety was greater than depression and stress and was frequently presented among women university students. Likewise, in another study developed by Hamaideh and Hamdan-Mansour [21] in health science students that included students of nursing found that anxiety was presented at a moderate level and that women suffered it more unlike men. In the study of Abebe et al. [20], it was found that anxiety (23.6%) was much higher than the percentages found for stress (4.1%) and depression (19.7%); likewise, Cheung et al. [14] also found higher values of anxiety (39.9%) compared to depression and stress.

In relation to stress, most nursing students have levels of normality; however, mild and moderate values are found in those between 20 and 29 years old, women, single women, have no children, and are dedicated to studying only. These sociodemographic variables are not associated with stress. These results can be contrasted with Hamaideh and Hamdan-Mansour [21] who found in their results that stress was presented at a mild level. In addition, according to the study by Shamsuddin et al. [15], it was also found that there was more depression and anxiety among the students. Otherwise, Abebe et al. [20] found only 4.1% prevalence of stress, while those of authors differ from the results found by Cheung et al. [14] who claimed that there was only 20% stress level in Chinese nursing students, as well as the study of Hamaideh and Hamdan-Mansour [21] who demonstrated 22.6% stress in Jordanian students. 

Through the results of this research, strategies and decision‐making can be selected against the health problems found in nursing students, whose unhealthy lifestyles lead to deterioration of their health and the appearance of noncommunicable diseases, especially in developing countries where mental health policies are not clear. The health behaviors found are also associated with sex. Therefore, it is important to keep in mind that the majority of the population in the Nursing School is made up of women, who by nature are exposed to hormonal changes of their gender, affecting not only their personal life but also academic aspects.

Alterations of mental nature are increasing, and young women are the most affected. This establishes a social problem where the presence of gender would imply the creation of programs considering these risk factors. Within the main imitations, it could be indicated that being a cross-sectional study, it would not allow knowing if there are changes in the levels of depression, anxiety, or stress, during the duration of the academic semester. Finally, the importance of this study consisted in determining lifestyles, depression, anxiety, and stress in the students of the Professional School of Nursing to make timely detection of problems that could affect their academic activity and thus be able to establish strategies for health prevention by promoting healthy practices among university students.

## 5. Conclusions

In conclusion, according to this logistic regression analysis, it was found that nursing students had unhealthy lifestyles and had moderate to extremely severe levels of anxiety, while most of them had normal levels of depression and stress. The majority of nursing students do not suffer from depression; however, there was a significant association between age and the presence of depression levels, as well as being a risk factor for this type of mental disorder. Finally, the students do not suffer from stress, and also no associations or risk factors were found regarding age, sex, marital status, number of children, and occupation.

## Figures and Tables

**Table 1:** Lifestyles, depression, anxiety, and stress in students of the Professional School of Nursing in Lima, Peru.

	*f*	%
*Lifestyle*		
Not healthy	643	53.9
Healthy	550	46.1
Normal	730	61.2
Mild	169	14.2

*Depression*		
Moderate	235	19.7
Severe	30	2.5
Extremely severe	29	2.4
Normal	265	22.2
Mild	286	24.0

*Anxiety*		
Moderate	433	36.3
Severe	144	12.1
Extremely severe	65	5.4
Normal	715	59.9
Mild	166	13.9

*Stress*		
Moderate	174	14.6
Severe	122	10.2
Extremely severe	16	1.3
Total	**1193**	**100**

**Table 2:** Risk factors of the students of the Professional School of Nursing according to the lifestyle.

Risk factors	Lifestyle						*p*	OR	CI 95%
	Unhealthy		Healthy		Total				
	*f*	%	*f*	%	*f*	%			
*Age*									
<20 years	239	20.0	202	16.9	441	37.0	0.839	1.1	0.7–1.7
20 to 29	354	29.7	300	25.1	654	54.8			
30 to 39	41	3.4	39	3.3	80	6.7			
40 to 49	8	0.7	9	0.8	17	1.4			
50 to 59	1	0.1	0	0.0	1	0.1			

*Sex*									
Female	572	47.9	456	38.2	1028	86.2	0.003	1.6	1.1–2.3
Male	71	6.0	94	7.9	165	13.8			
Single	560	46.9	479	40.2	1039	87.1			

*Civil status*									
Married	29	2.4	31	2.6	60	5.0	0.632	0.9	0.6–1.3
Cohabitants	45	3.8	31	2.6	76	6.4			
Separated	9	0.8	9	0.8	18	1.5			
None	537	45.0	466	39.1	1003	84.1			

*Number of children*									
1 to 2	94	7.9	74	6.2	168	14.1	0.827	0.9	0.6–1.2
3 to 4	10	0.8	7	0.6	17	1.4			
4 or more	2	0.2	3	0.3	5	0.4			

*Occupation*									
Just study	412	34.5	325	27.5	737	61.8	0.077	1.2	0.9–1.5
Study and work	231	19.4	225	18.9	456	38.2			

**Table 3:** Risk factors of the students of the Professional School of Nursing according to the level of depression.

Risk factors	Depression												*p*	OR	CI 95%
	Normal		Mild		Moderate		Severe		Ex. Severe		Total				
	*f*	%	*f*	%	*f*	%	*f*	%	*f*	%	*f*	%			
*Age*															
<20 years	273	22.9	68	5.7	77	6.5	8	0.7	15	1.3	441	37.0	0.040	2.0	1.3–3.1
20 to 29	413	34.6	83	7.0	127	10.6	18	1.5	13	1.1	654	54.8			
30 to 39	36	3.0	15	1.3	25	2.1	4	0.3	0	0.0	80	6.7			
40 to 49	8	0.7	3	0.3	5	0.4	0	0.0	1	0.1	17	1.4			
50 to 59	0	0.0	0	0.0	1	0.1	0	0.0	0	0.0	1	0.1			

*Sex*															
Female	629	52.7	145	12.2	201	16.8	28	2.3	25	2.1	1028	86.2	0.846	0.9	0.7–1.4
Male	101	8.5	24	2.0	34	2.8	2	0.2	4	0.3	165	13.8			
Single	639	53.6	150	12.6	201	16.8	22	1.8	27	2.3	1039	87.1			

*Civil status*															
Married	29	2.4	11	0.9	15	1.3	4	0.3	1	0.1	60	5.0	0.265	1.1	0.8–1.6
Cohabitants	50	4.2	6	0.5	15	1.3	4	0.3	1	0.1	76	6.4			
Separated	12	1.0	2	0.2	4	0.3	0	0.0	0	0.0	18	1.5			
None	618	51.8	145	12.2	189	15.8	23	1.9	28	2.3	1003	84.1			

*Number of children*															
1 to 2	98	8.2	22	1.8	40	3.4	7	0.6	1	0.1	168	14.1	0.222	1.1	0.8–1.5
3 to 4	13	1.1	1	0.1	3	0.3	0	0.0	0	0.0	17	1.4			
4 or more	1	0.1	1	0.1	3	0.3	0	0.0	0	0.0	5	0.4			

*Occupation*															
Just study	455	38.1	107	9.0	141	11.8	13	1.1	1.8	737	61.8	61.8	0.179	1.0	0.8–1.3
Study and work	275	23.1	62	5.2	94	7.9	17	1.4	8	0.7	456	38.2			

**Table 4:** Risk factors of the students of the Professional School of Nursing according to the level of anxiety.

Risk factors	Anxiety												*p*	OR	CI 95%
	Normal		Mild		Moderate		Severe		Ex. Severe		Total				
	*f*	%	*f*	%	*f*	%	*f*	%	*f*	%	*f*	%			
*Age*															
<20 years	81	6.8	122	10.2	163	13.7	55	4.6	20	1.7	441	37.0	0.140	0.9	0.6–1.6
20 to 29	162	13.6	138	11.6	232	19.4	79	6.6	43	3.6	654	54.8			
30 to 39	18	1.5	22	1.8	29	2.4	10	0.8	1	0.1	80	6.7			
40 to 49	3	0.3	4	0.3	9	0.8	0	0.0	1	0.1	17	1.4			
50 to 59	1	0.1	0	0.0	0	0.0	0	0.0	0	0.0	1	0.1			

*Sex*															
Female	240	20.1	238	19.9	371	31.1	120	10.1	59	4.9	1028	86.2	0.072	**1.7**	**1.0**–**2.6**
Male	25	2.1	48	4.0	62	5.2	24	2.0	6	0.5	165	13.8			
Single	230	19.3	256	21.5	374	31.3	119	10.0	60	5.0	1039	87.1			

*Civil status*															
Married	11	0.9	18	1.5	21	1.8	8	0.7	2	0.2	60	5.0	**0.043**	0.9	0.6–1.4
Cohabitants	20	1.7	9	0.8	27	2.3	17	1.4	3	0.3	76	6.4			
Separated	4	0.3	3	0.3	11	0.9	0	0.0	0	0.0	18	1.5			
None	225	18.9	246	20.6	353	29.6	120	10.1	59	4.9	1003	84.1			

*Number of children*															
1 to 2	37	3.1	36	3.0	67	5.6	22	1.8	6	0.5	168	14.1	0.588	1.0	0.7–1.5
3 to 4	2	0.2	4	0.3	10	0.8	1	0.1	0	0.0	17	1.4			
4 or more	1	0.1	0	0.0	3	0.3	1	0.1	0	0.0	5	0.4			

*Occupation*															
Just study	158	13.2	181	15.2	271	22.7	86	7.2	41	3.4	737	61.8	0.875	0.8	0.6–1.1
Study and work	107	9.0	105	8.8	162	13.6	58	4.9	24	2.0	456	38.2			

**Table 5:** Risk factors of the students of the Professional School of Nursing according to the level of stress.

Risk factors	Stress												*p*	OR	CI 95%
	Normal		Mild		Moderate		Severe		Ex. Severe		Total				
	*f*	%	*f*	%	*f*	%	*f*	%	*f*	%	*f*	%			
*Age*															
<20 years	266	22.3	55	4.6	65	5.4	48	4.0	7	0.6	441	37.0	0.904	0.8	0.5–1.2
20 to 29	386	32.4	100	8.4	95	8.0	65	5.4	8	0.7	654	54.8			
30 to 39	49	4.1	11	0.9	10	0.8	9	0.8	1	0.1	80	6.7			
40 to 49	13	1.1	0	0.0	4	0.3	0	0.0	0	0.0	17	1.4			
50 to 59	1	0.1	0	0.0	0	0.0	0	0.0	0	0.0	1	0.1			

*Sex*															
Female	618	51.8	144	12.1	145	12.2	106	8.9	15	1.3	1028	86.2	0.720	1.0	0.7–1.4
Male	97	8.1	22	1.8	29	2.4	16	1.3	1	0.1	165	13.8			
Single	619	51.9	147	12.3	154	12.9	105	8.8	14	1.2	1039	87.1			

*Civil status*															
Married	36	3.0	5	0.4	10	0.8	8	0.7	1	0.1	60	5.0	0751	10	07–1.4
Cohabitants	45	38	13	11	10	08	7	06	1	01	76	64			
Separated	15	1.3	1	0.1	0	0.0	2	0.2	0	0.0	18	1.5			
None	592	49.6	138	11.6	151	12.7	107	9.0	15	1.3	1003	84.1			

*Number of children*															
1 to 2	106	8.9	26	2.2	20	1.7	15	1.3	1	0.1	168	14.1	0.428	0.7	0.5–1.0
3 to 4	15	1.3	1	0.1	1	0.1	0	0.0	0	0.0	17	1.4			
4 or more	2	0.2	1	0.1	2	0.2	0	0.0	0	0.0	5	0.4			

*Occupation*															
Just study	443	37.1	104	8.7	105	8.8	76	6.4	9	0.8	737	61.8	0.979	1.0	0.8–1.2
Study and work	272	22.8	62	5.2	69	5.8	46	3.9	7	0.6	456	38.2			
